# Application of FTIR to detect changes in blood plasma during a pre-competition period in a professional heavyweight boxer

**DOI:** 10.3389/fphys.2026.1845333

**Published:** 2026-10-19

**Authors:** Paweł Król, Joanna Depciuch, Adam Reich, Józef Cebulski, Zbigniew Obmiński, Krzysztof Kasicki, Wojciech Czarny, Łukasz Różański, Łukasz Rydzik

**Affiliations:** 1 Faculty of Physical Culture Sciences, College of Medical Sciences, University of Rzeszow, Rzeszow, Poland; 2 Institute of Nuclear Physics, Polish Academy of Sciences, Kraków, Poland; 3 Department of Biochemistry and Molecular Biology, Medical University of Lublin, Lublin, Poland; 4 Department of Dermatology, Faculty of Medicine, Collegium Medicum, University of Rzeszow, Rzeszow, Poland; 5 Institute of Physics, Faculty of Exact and Technical Sciences, University of Rzeszow, Rzeszow, Poland; 6 Department of Endocrinology, Institute of Sport-National Research Institute, Warsaw, Poland; 7 Department of Sport Theory and Motor Skills, Institute of Sport Sciences, University of Physical Culture in Kraków, Kraków, Poland; 8 Faculty of Sports, University of Presov, Prešov, Slovakia

**Keywords:** ATR-FTIR spectroscopy, biomarker monitoring, blood plasma, combat sports, competition, single-case study, tapering

## Abstract

**Objectives and design:**

This exploratory single-case study examined whether repeated attenuated total reflectance Fourier transform infrared (ATR-FTIR) spectroscopy of blood plasma is technically feasible during a short pre-competition period and whether spectral regions co-vary with conventionally assayed biochemical markers.

**Methods:**

One professional heavyweight boxer was monitored over a 13-day pre-competition period characterized by reduced training load (tapering). Morning fasting blood was collected on five occasions (10, 13, 17, 20, and 23 May 2024) into lithium-heparin tubes and centrifuged to obtain plasma. Creatine kinase (CK), C-reactive protein (CRP), glucose, cortisol, testosterone, total and reduced glutathione, malondialdehyde, and zinc were quantified by conventional laboratory assays; ATR-FTIR spectra were acquired separately from the same plasma aliquots and were not used to quantify any of these analytes. Each plasma sample was measured eight times, yielding 40 spectra from five biological observations; the technical replicates are presented to illustrate within-sample visual consistency. Associations between the intensities of 22 spectral peaks and 12 biochemical variables were explored using Pearson correlation.

**Results:**

Plasma biochemical parameters showed changes during the pre-competition tapering period, including decreases in CK and CRP and increases in glutathione and testosterone, whereas cortisol remained relatively stable. FTIR spectra exhibited consistent vibrational profiles with changes in selected band intensities over time. Linear regression identified strong associations between glutathione levels and FTIR bands at 671, 699, and 721 cm^−1^ (*R*^2^ = 0.80–0.92), as well as between cortisol and the 3,009 cm^−1^ band (*R*^2^ = 0.77). Because of the limited number of observations (*n* = 5), these findings are preliminary and require validation in larger cohorts.

**Conclusions:**

Repeated plasma measurements using ATR-FTIR demonstrated technical feasibility and visual consistency during a short pre-competition period in the studied athlete, while identifying potential spectral regions worthy of further investigation. The obtained results should be treated as preliminary. However, they do not provide evidence that ATR-FTIR enables the quantitative determination of glutathione or cortisol, or that it can be used to assess an athlete’s competitive readiness. Confirmation of such capabilities requires studies involving adequately large and diverse groups of participants.

## 1 Introduction

Monitoring resting changes in selected biochemical markers in the blood plays an important role in assessing the tolerance of physical efforts during the preparation period for competition among competitive athletes. Biochemical status is not stable but fluctuates from day to day, and it depends on the magnitude of the training load and other stimuli applied on the days before the testing. A change in each indicator may indicate a disturbance in homeostasis in a specific system or tissue. There are many groups of indicators used in sport medicine, and each of them is responsible for the functional state of the proper system. For example, exercise-induced muscle damage manifests itself as an increase in the levels of cytosolic enzymes in the blood. A study by Clarkson showed that 50 maximal eccentric contractions of the elbow flexor muscles brought about an increase of muscle enzyme levels in the circulation like creatine kinase (CK), myoglobin (Mb), lactate dehydrogenase (LDH), alanine aminotransferase (ALT), and aspartate aminotransferase (AST), but the highest change was noted for CK (Clarkson et al., 2006). For this reason, CK seems to be the most sensitive marker of muscle damage, and CK measurements have long been used in sports medicine (Brancaccio et al., 2007). In order to determine the reference intervals for the CK, a large group of athletes of both sexes was examined during training and/or competition. Men showed CK levels twice as high as those of women, and a very wide range of values of the enzyme were recorded, min–max: 82–1,083 U/L (Mougios, 2007). However, not only different physical stimuli are responsible for such diverse values of CK. Large inter-individual differences in response to the same type of exercise were demonstrated by several authors (Hagstrom and Shorter, 2018); therefore, it allows classifying athletes as high or low responders. The post-exercise CK peak of the high-responding athlete group averaged 751 U/L and was significantly higher than the corresponding value (184 U/L) recorded in the low-responding group (Totsuka et al., 2002). The other study indicated that CK peak was positively correlated with the relative load of the working muscles. High responders demonstrated lower workload/cross-sectional area quadriceps femoris muscles and lower mobilization of free fatty acids as compared to those of low responders. Moreover, the work volume, the mass of the involved muscles, and various types of muscle contractions (eccentric or concentric) contributed to the magnitude of CK response to exercise, and perceived muscle soreness are higher in high responders (Brancaccio et al., 2010). Interestingly, the link between magnitude of exertional CK and a marker of inflammation, C-reactive protein (CRP), was shown. Post-exercise plasma CK was much higher when CRP responses were also higher. In addition, in high responders, elevated CK was observed up to 4 days after exertion, while in low responders, the enzyme level normalized on the second day (Isaacs et al., 2019).

There are many symptoms associated with exercise-induced muscle damage like delayed soreness of active limbs, drop of physical performance, and post-exercise inflammation processes that manifest itself as changes in proinflammatory cytokines like interleukin-6 (IL-6), interleukin-8 (IL-8), tumor necrosis factor-alpha (TNF-α), and CRP. Assessments of plasma CK, TNF-α, and CRP are especially recommended in the long term. Investigations on these indicators returning to their initial values during post-exercise recovery are of high diagnostic value (Tota et al., 2019).

Based on the mentioned plasma parameters, some authors recommend special diet and supplements to enhance post-training recovery (Markus et al., 2021).

Observation of hormonal responses provides an additional tool that is utilized for testing of metabolic state during a training period and psycho-emotional state prior to a competition. Levels of steroid hormones, cortisol (C) and testosterone (T), in plasma are most often used for the assessment of the so-called anabolic–catabolic index that is expressed as T-to-C ratio, with the levels of these hormones expressed in nanomolar. Usually, the index is measured in the morning under fasting conditions or after post-exercise state to establish the current rate of endogenous protein metabolism (Brini et al., 2020; De Luccia, 2016; Puiu et al., 2019; Sollberger and Ehlert, 2016).

Some authors point out different directions of changes pre–post competition in cortisol and testosterone levels. Most of the studies have shown that athletes with higher cortisol and testosterone during competition achieve better performance (Jiménez et al., 2012; Papacosta et al., 2016). Investigations of the hormonal status in blood or saliva showed a significantly higher cortisol level before a competition as compared to that under neutral conditions (Ficarra et al., 2023). That discrepancy is an effect of anticipation of stressful events. The symptoms of high pre-competitive stress manifest themselves as both higher cortisol levels and scores of state anxiety as compared to these indicators observed under neutral conditions (Capranica et al., 2017; Salvador et al., 2003; Souza et al., 2019). Moreover, the interplay between the two hormones is worth noticing. Induced by endurance exercise, a very high cortisol level inhibits the release of testosterone into circulation in men. It manifests itself as a negative post-exercise correlation between both hormones during post-exertion recovery (Daly et al., 2005).

Another important group of indicators of an exercise response is related to the system of antioxidant defense and signaling of so-called oxidative stress. It is useful in monitoring both exercise tolerance and health in athletes. Several chemical compounds in blood play an important role in reducing the harmful effect of oxidative stress, by eliminating free radicals, the mainly reactive form of oxygen formed as a result of aerobic metabolism of proteins and lipids. The most effective natural antioxidants are total bilirubin, uric acid, coenzyme Q10, tocopherols, retinol, carotenoids, vitamin C, and reduced form of glutathione.

This exploratory study investigates the feasibility of attenuated total reflectance Fourier transform infrared (ATR-FTIR) measurements of blood plasma during a pre-competition tapering period in a professional heavyweight boxer. The study further explores whether temporal changes in selected biochemical parameters are reflected in corresponding changes in plasma FTIR spectral bands. Given the single-participant design and the limited number of biological observations, the observed spectral–biochemical associations are considered hypothesis-generating rather than confirmatory. None of the identified correlations remained statistically significant after correction for multiple testing, and no validated predictive model or diagnostic or predictive capability is claimed. The eight technical replicates acquired per sample were obtained under identical analytical conditions and are presented to illustrate within-sample visual consistency; they do not establish quantitative repeatability or reproducibility across independent runs, operators, instruments, or measurement days. The primary contribution of the study is therefore the assessment of the technical feasibility and within-sample visual consistency of plasma FTIR measurements under real-world conditions, together with the identification of spectral regions that may warrant further investigation in larger and adequately powered studies.

## 2 Methods

### 2.1 Study design and reporting

This study is a prospective and exploratory single-case study (*n* = 1) with five repeated biological observations. Reporting follows the CARE guidelines for case reports, extended with analytical chemistry reporting items specific to vibrational spectroscopy. The study was designed as a methodological feasibility and hypothesis-generating exercise; no *a priori* hypotheses were tested and no sample size calculation was performed, as they are not justified in single-case designs. Throughout the article, the term “biological observation” denotes one blood collection (*n* = 5) and “technical replicate” denotes one of the eight repeated spectral acquisitions performed on a single plasma sample (*n* = 40 spectra).

### 2.2 Case study participant

One male professional boxer competing in the heavyweight division was monitored over a 13-day period preceding a scheduled bout against a highly ranked opponent. At study entry, the participant was 38 years old, 186 cm tall, and weighed 103.7 kg, with 11 years of professional boxing experience and 16 bouts fought at this level. Body mass changed by −1.6 kg between the first and last collection, from 103.7 to 102.1 kg. The athlete reported no acute illness, injury, or infection during the observation period or in the preceding 4 weeks; was taking no prescription medication; and used a vitamin D3 preparation and a whey protein supplement, both maintained at a constant dose throughout the period. No rapid weight-loss or dehydration procedures were applied during the observation window. Blood was collected on five occasions (10, 13, 17, 20, and 23 May 2024) at 07:30, after an overnight fast of at least 10 h, before the first training session of the day and at least 14 h after the completion of the preceding session, with the participant seated and rested for 15 min prior to venipuncture. Tubes were centrifuged at 2,000 × *g* for 10 min at 4 °C within 30 min of collection; plasma was aliquoted and stored at −80°C until analysis. All biochemical assays and spectroscopic measurements were performed in a single analytical run after the end of the observation period. Samples underwent a single freeze**–**thaw cycle. The participant gave written informed consent to take part in the study and, separately, written informed consent for the publication of the presented data, having been advised that this information may render him identifiable. The study was conducted in accordance with the Declaration of Helsinki and was approved by the Bioethics Committee of the University of Rzeszów (protocol code 8/11/2020, dated 8 December 2020).

### 2.3 Training load during the observation period

Training was planned by the athlete’s coaching staff and prospectively recorded in the training log. This period corresponded to the final stage of tapering: total weekly volume was gradually reduced, while the intensity of technical work was maintained. The rating of perceived exertion (sRPE, CR-10) multiplied by session duration was used to express daily internal load in arbitrary units (AU) (**Table 1**). Sleep quality scores on D1, D2, D3, D4, and D5 remained almost unchanged (3, 3, 3, 2, and 3, respectively).

**Table 1:** A summary of the load in the 48 h preceding each blood collection.

Collection	Date	Day of study	Training volume in the preceding 48 h (min)	Internal load in the preceding 48 h (AU)	Sparring rounds in the preceding 48 h	Time since last session (h)
D1	10 May 2024	0	195	1,310	6	14
D2	13 May 2024	3	170	1,080	4	15
D3	17 May 2024	7	140	760	2	16
D4	20 May 2024	10	105	520	0	18
D5	23 May 2024	13	60	240	0	20

### 2.4 Reference biochemical assays

The concentrations of biochemical parameters reported in this article were determined using conventional laboratory assays performed independently of, and in parallel with, the spectroscopic measurements. FTIR was not used for the quantitative determination of any of these analytes; the laboratory assay results served as reference values against which the spectral data were examined for co-variation. All assays were performed within a single analytical run. Each sample was assayed in duplicate, and the mean of the two measurements was used for subsequent analyses. Within-run imprecision (CV_A_) was determined from eight consecutive measurements of a single pooled plasma sample performed within the same analytical run. Oxidized glutathione was not measured directly but was calculated as [total glutathione (TGlu) − reduced glutathione (RGlu)]/2. Accordingly, its imprecision, as well as that of the RGlu/TGlu and RGlu/OxGlu ratios, was determined by propagation of the uncertainties of the component measurements.

CK, CRP, glucose, cortisol, testosterone, and zinc were determined at Centrum Medyczne Medyk, Laboratory Diagnostics Department, Rzeszów, Poland, a medical diagnostic laboratory operating in accordance with the applicable Polish regulations governing laboratory diagnostics. CK, CRP, and glucose were measured on an Alinity c clinical chemistry analyzer, and cortisol and testosterone were measured on an Alinity i immunoassay analyzer (both Abbott Laboratories, Abbott Park, IL, USA), using the manufacturer’s reagent kits and calibrators listed in **Table 2**. Zinc was determined by inductively coupled plasma mass spectrometry on an ICPMS-2040 LF instrument (Shimadzu Corporation, Kyoto, Japan). Calibration was verified before each analytical run, and internal quality control was performed at two concentration levels within each run. All quality-control results were within the acceptance limits specified by the manufacturer.

**Table 2:** Measurement platforms, reagents, and within-run analytical imprecision (CVₐ) of the reference biochemical assays.

Test	Analyzer model	Reagent name (catalogue no.)	Manufacturer	CV_A_ (%)
Creatine kinase	Alinity c	CK Reagent Kit (08P)	Abbott	1.3
C-reactive protein	Alinity c	CRP Vario Reagent Kit (07P5620)	Abbott	0.9
Glucose	Alinity c	Glucose Reagent Kit (07P5530)	Abbott	0.9
Cortisol	Alinity i	Cortisol Reagent Kit (08P3320)	Abbott	1.2
Testosterone	Alinity i	Testosterone II Reagent Kit (07P6822)	Abbott	1.2
Zinc	ICPMS-2040 LF	—	Shimadzu	0.3
Total glutathione	CLARIOstar Plus	Glutathione Assay Kit (CS0260)	Sigma-Aldrich	0.8
Reduced glutathione	CLARIOstar Plus	GSH Assay Kit (MAK440)	Sigma-Aldrich	0.9
Malondialdehyde	CLARIOstar Plus	Lipid Peroxidation (MDA) Assay Kit (MAK085)	Sigma-Aldrich	0.4
Oxidized glutathione	Calculated: (total − reduced glutathione)/2	—	—	0.7
Testosterone-to-cortisol ratio × 100	Calculated	—	—	1.70
Reduced-to-total glutathione ratio	Calculated	—	—	1.20
Reduced-to-oxidized glutathione ratio	Calculated	—	—	1.14

TGlu and RGlu and malondialdehyde were determined using the Sigma-Aldrich assay kits identified in **Table 2** (Merck KGaA, Darmstadt, Germany), read on a CLARIOstar Plus microplate reader (BMG LABTECH, Ortenberg, Germany), with a matrix-matched calibration curve prepared within each analytical run. A broader panel of routine laboratory tests was performed on the same samples as part of the athlete’s standard medical monitoring; those results are not reported in this article, and only the assays underlying the reported variables are listed in **Table 2**.

### 2.5 FTIR spectroscopy measurement

Plasma samples were thawed at room temperature and gently mixed before FTIR analysis. For sample preparation, 4 µL of plasma was deposited onto a CaF_2_ window and allowed to dry at room temperature for approximately 10 min. The CaF_2_ windows were used as substrates for sample deposition and drying, allowing multiple samples to be prepared and dried in parallel prior to spectral acquisition. After complete drying, each CaF_2_ window containing the dried plasma sample was placed directly onto the diamond ATR crystal of the Nicolet iS50 FTIR spectrometer for spectral acquisition. Thus, the CaF_2_ window was used only for sample preparation and drying, whereas the diamond crystal served as the ATR sensing element during the FTIR measurement. Before sample measurements, a background spectrum was collected. FTIR spectra were recorded in the range of 400–4,000 cm^−1^ at a spectral resolution of 4 cm^−1^ using 32 scans per spectrum. Each sample was measured eight times, resulting in 40 spectra in total. Between consecutive measurements, the diamond ATR crystal was cleaned with 70% ethanol and thoroughly dried. The acquired spectra were processed using OPUS 7.0 software. Preprocessing included Rubberband baseline correction and vector normalization. Subsequently, the eight spectra acquired for each sample were averaged to obtain a representative mean spectrum for each sample. All spectra are presented in **Supplementary Figures S1**–**S5**.

### 2.6 Data analysis, Pearson correlation test, linear regression model, and between-day comparison of the biochemical parameters

To obtain information on the correlation between FTIR data and CK, CRP, glucose, cortisol, testosterone, TGlu, RGlu, oxidized glutathione, RGlu/TGlu ratio, RGlu/OxGlu ratio, malondialdehyde, and zinc concentrations, the Pearson correlation test was performed using Past 3.0 software. For this purpose, the FTIR intensity of the peaks described in the obtained spectra was used and correlated with the biochemical parameters (uncorrected *p* < 0.05). Peaks, which showed even nominal correlation with parameters obtained from laboratory tests, were used to build a linear regression model. For this purpose, the ordinary least squares method was used to obtain a fitted curve pattern. In addition, the 95% confidence bands of the regression lines were computed. Because the intensities of 22 spectral bands were correlated with 12 biochemical variables, the spectral–biochemical analysis comprised 264 tests, and the correlations computed among the biochemical variables themselves comprised a further 66 tests. To control the expected proportion of false-positive findings, *p*-values were adjusted within each family using the Benjamini–Hochberg false discovery rate procedure, and adjusted values are denoted *q*. No association reached *q* < 0.05 in either family. Uncorrected *p*-values are therefore reported descriptively, and all associations are described as nominal rather than statistically significant.

## 3 Results

A professional heavyweight boxer was examined to determine whether the variability of assayed stress-related parameters is accompanied by variation in the FTIR spectrum. Concentrations of CK, CRP, glucose (G), cortisol (C), testosterone (T), TGlu, RGlu, oxidized glutathione (OxGlu), the ratios of RGlu to TGlu and RGlu to OxGlu, malondialdehyde (MDA), and zinc were determined using the laboratory assays described in the Methods section on 5 days of the pre-competition period (10, 13, 17, 20, and 23 May 2024). As shown in **Table 3**, the parameters fluctuated during this period, and some of them exhibited a directional pattern. Comparing the first collection (May 10) with the last (May 23), the levels of glucose, TGlu, RGlu, testosterone, and the T-to-C ratio were higher at the end of the period, while the levels of CK and CRP were lower. The corresponding slopes of the line fitted using the least squares method for all five collections are presented in **Table 3**. None of the corresponding 95% confidence intervals excludes zero, with the exception of TGlu (41.0 µmol·L^-1^·day^-1^, 95% CI 1.2 to 80.8, *p* = 0.046, *R*^2^ = 0.78). Between-day variability, expressed as the coefficient of variation, was largest for the RGlu-to-OxGlu ratio (133.9%), OxGlu (56.9%), CRP (43.3%), CK (37.6%), and RGlu (36.4%), and smallest for cortisol (4.4%) and glucose (6.4%).

**Table 3:** Variability (CV%) in plasma parameters across the short training period prior to the competition, with the ordinary least-squares slope fitted across all five collections.

Parameter	10 May	13 May	17 May	20 May	23 May	CV%	Slope	Intercept
CK (U/L)	495	257	356	281	183	37.6	−17.61	430.6
CRP (mg/dL)	0.27	0.15	0.34	0.16	0.13	43.3	−0.0074	0.259
G (mg/dL)	82	82	79	89	92	6.4	0.793	79.6
C (nmol/L)	451	481	448	462	426	4.4	−2.123	467.6
T (nmol/L)	15.23	18.18	14.94	16.24	17.55	8.6	0.069	15.97
T-to-C-ratio × 100	3.4	3.8	3.3	3.5	4.1	9.0	0.031	3.42
TGlu (µmol/L)	983	931	1,400	1,364	1,425	19.9	41.01	949.9
RGlu (µmol/L)	553	663	1,328	793	1,125	36.4	40.21	627.0
OxGlu (µmol/L)	215	134	36	286	150	56.9	0.416	161.5
RGlu/TGlu	0.56	0.71	0.95	0.58	0.79	22.4	0.0106	0.648
RGlu/OxGlu	2.6	4.9	36.9	2.8	7.5	133.9	0.343	8.67
MDA (µmol/L)	0.73	0.81	0.62	0.72	0.79	10.1	0.0002	0.733
Zinc (µg/dL)	86	116	80	90	89	15.0	−0.711	96.9

CV% = coefficient of variation across the five observations, calculated as (standard deviation with *n* − 1 in the denominator ÷ arithmetic mean) × 100. Slope = ordinary least-squares slope fitted across all five collections time in days from baseline (0, 3, 7, 10, and 13), given per day.

Pairwise Pearson linear correlations between the 12 biochemical parameters were examined. The strongest associations were observed between T and MDA (*r* = 0.886, 95% CI 0.02 to 0.99, *p* = 0.045), CRP and T (*r* = −0.877, 95% CI −0.99 to 0.02, *p* = 0.051), CRP and MDA (*r* = −0.866, 95% CI −0.99 to 0.06, *p* = 0.058), CRP and glucose (*r* = −0.803, 95% CI −0.99 to 0.25, *p* = 0.102), CRP and the RGlu-to-OxGlu ratio (*r* = 0.769, 95% CI −0.33 to 0.98, *p* = 0.128), and CRP and CK (*r* = 0.741, 95% CI −0.39 to 0.98, *p* = 0.152). After applying the Benjamini**–**Hochberg correction for 66 tests, no association reached the level of *q* < 0.05. Therefore, the coefficients are presented solely to document the internal structure of the dataset and cannot be attributed to training load.

FTIR spectra were collected from the same plasma samples for which levels of parameters presented in **Table 3** were obtained. **Figure 1** shows that, in each plasma sample, the same vibrations were visible. Peaks at 671 and 699 cm^−1^ corresponded to C–H out-of-plane aromatic bands (Barth, 2007; Movasaghi et al., 2008). Vibrations of N–H groups from thymine were visible at 721 and 797 cm^−1^ (Movasaghi et al., 2008; Talari et al., 2017). Peaks at 846 and 929 cm^−1^ originated from stretching vibrations of C–C from nucleic acid (Movasaghi et al., 2008). Stretching vibrations of PO_2_^−^ groups were observed at 1,024 and 1,260 cm^−1^, while the peak at 1,164 cm^−1^ originated from CC–OC vibrations from polysaccharides (Talari et al., 2017). Amide vibrations were located at 1,376, 1,542, 1,642, and 3,272 cm^−1^ (Barth, 2007). Moreover, CH_2_ and CH_3_ vibrations for proteins and lipids were visible at 1,415 and 1,458 cm^−1^ (Talari et al., 2017). Finally, C=O, CH_2_, and CH_3_ lipid vibrations were visible at 1,711, 1,738, 2,853, 2,922, 2,957, 3,009, and 3,065 cm^−1^ (Talari et al., 2017).

**Figure 1:**
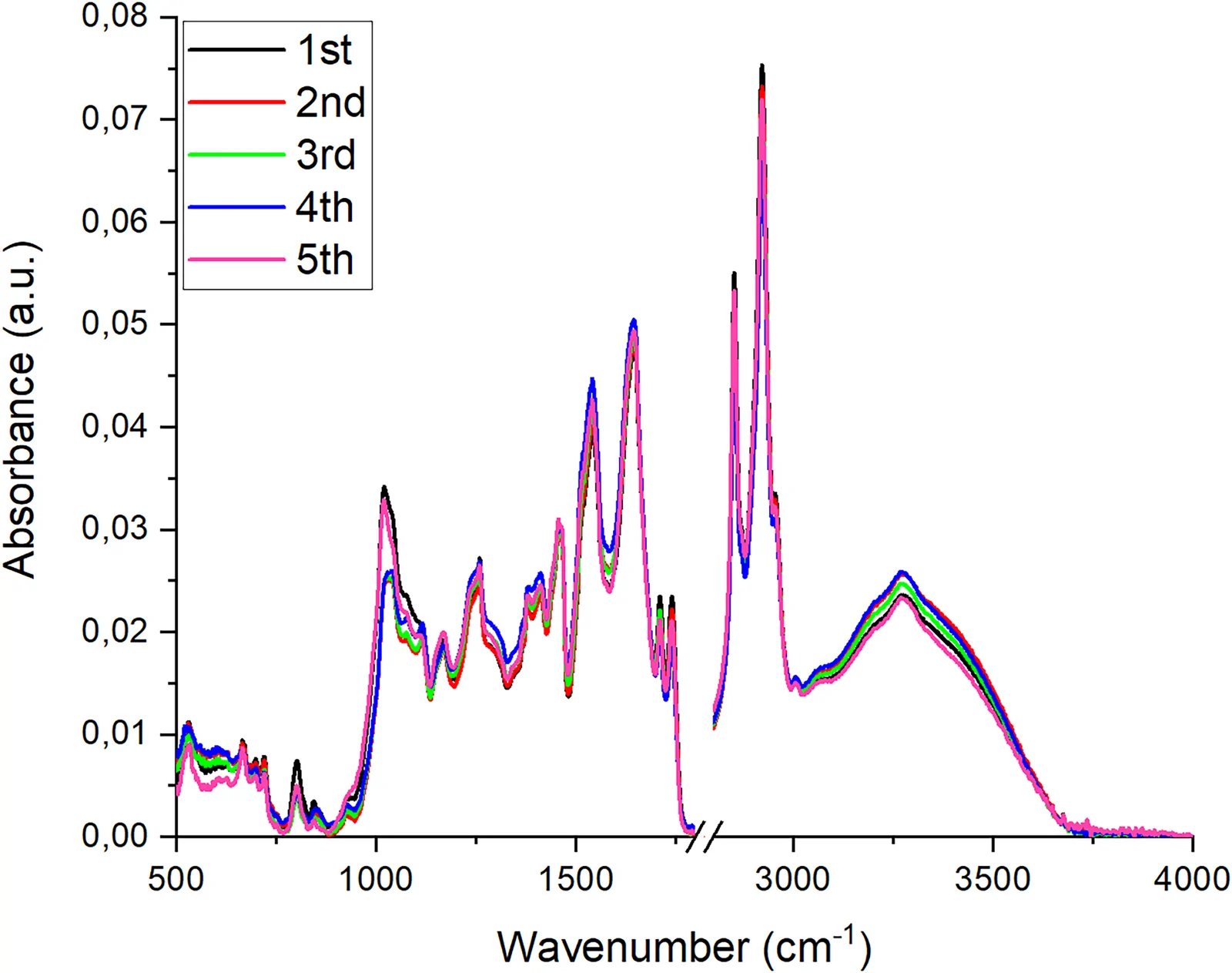
Mean FTIR spectra of plasma collected from professional heavyweight boxer on 10 May 2024 (1st), 13 May 2024 (2nd), 17 May 2024 (3rd), 20 May 2024 (4th), and 23 May 2024 (5th).

The largest between-sample differences in absorbance were observed for the band at 1,024 cm^-1^, for which relatively high values were recorded in the samples collected on 10 May 2024 and 23 May 2024. Differences between samples were also apparent at 3,272 cm^-1^, with the highest absorbance in the samples of 13 May 2024 and 20 May 2024 and the lowest on 10 May 2024. These differences are described visually and were not tested statistically. Because no quantitative measure of within-sample variability is reported, their magnitude should be interpreted in the light of the spread of the eight technical replicates shown in **Supplementary Figures S1**–**S5** (**Figure 1**).

Pearson correlation test performed for all obtained stress parameters and analyzed peaks visible in FTIR spectra showed that correlations between FTIR and laboratory data were observed only for cortisol and peak at 3,009 cm^−1^; TGlu and peaks at 671, 699, and 721 cm^−1^; and RGlu and peaks at 671 and 699 cm^−1^ (**Figure 2**). **Table 4** shows that in the cases of TGlu and RGlu, correlations between laboratory and FTIR data were negative, while those between cortisol and peak at 3,009 cm^−1^ were positive.

**Figure 2:**
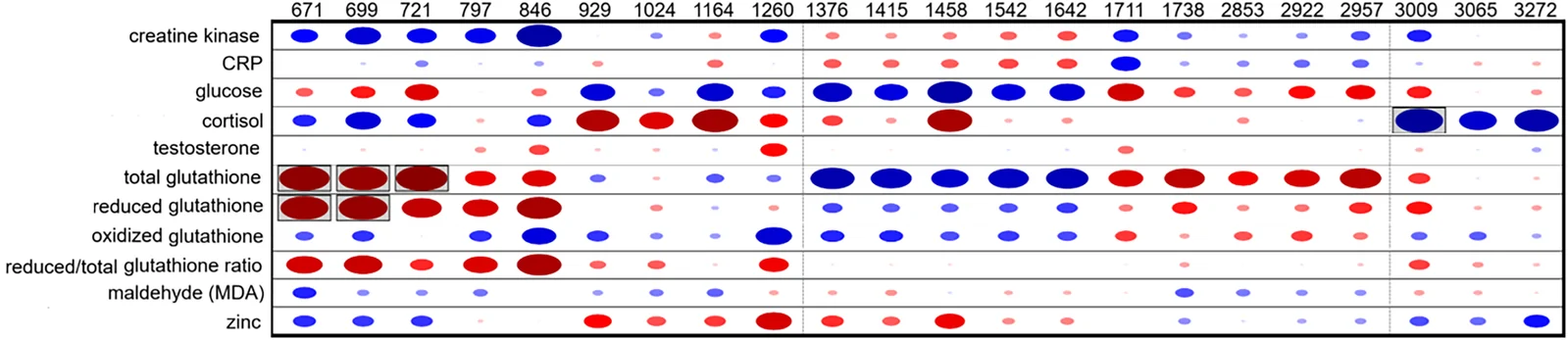
Pearson correlation test between medical parameters presented in **Table 3** and FTIR peaks.

**Table 4:** Pearson correlation test between the concentration of cortisol, total and reduced glutathione in plasma, and the absorbance of peaks analyzed in FTIR spectra.

Biochemical parameter	FTIR band (cm^-1^)	Pearson’s r
Total glutathione	671	–0.93
	699	–0.90
	721	–0.96
Reduced glutathione	671	–0.89
	699	–0.91
Cortisol	3,009	0.89

Pearson’s correlation coefficients are specified for *p* < 0.05.

The bands showing the strongest correlations with TGlu, RGlu, and cortisol were used to fit simple least-squares regression lines, shown in **Figure 3**. These lines are presented solely as an illustration of exploratory calibration. The bands were selected based on their correlations within the same five observations that were subsequently used to fit and evaluate the models.

**Figure 3:**
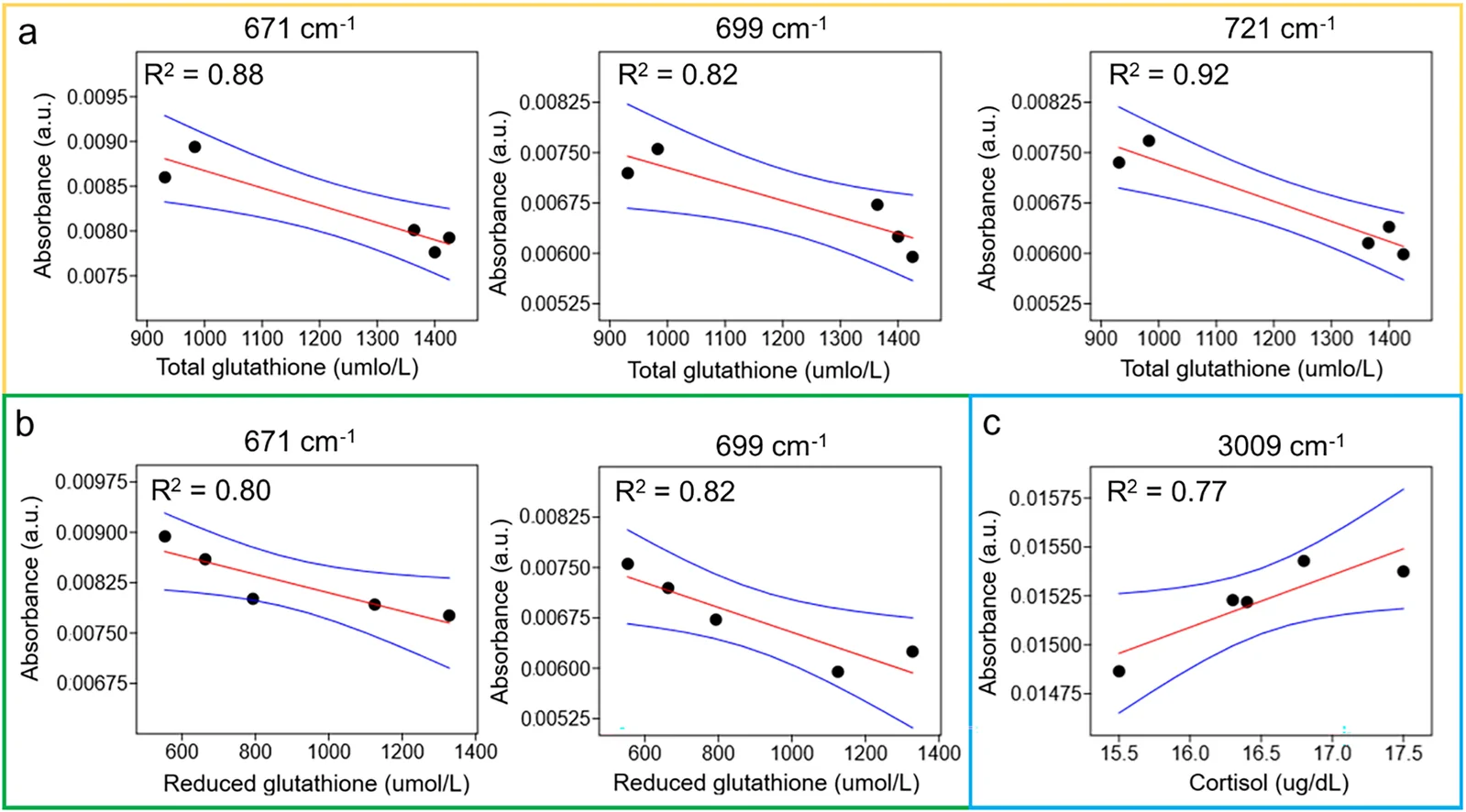
Linear-regression model for total glutathione **(a)**, reduced glutathione **(b)**, and cortisol **(c)**. Red line, ordinary least-squares fit; blue lines, 95% confidence band; R², in-sample coefficient of determination (n = 5).

The apparent in-sample *R*^2^ values were 0.92 (721 cm^−1^), 0.86 (671 cm^−1^), and 0.82 (699 cm^−1^) for TGlu; 0.82 (699 cm^−1^) and 0.80 (671 cm^−1^) for RGlu; and 0.77 (3,009 cm^−1^) for cortisol.

Between-day variability is reported descriptively in **Table 3** as the coefficient of variation across the five collections. The largest relative fluctuation was recorded for the glutathione fractions and their ratios, for CK and for CRP, and the smallest for cortisol and glucose. No threshold was applied to classify these differences as real or otherwise.

## 4 Discussion

The exertion to which the body is subjected during professional fights and training in professional boxing poses a challenge to the human system, combining the demand for anaerobic power—the ability to generate maximal muscle contractions in the shortest possible time—with the necessity of maintaining high aerobic capacity throughout several dozen minutes of combat. This dichotomy, coupled with the mechanical stress resulting from receiving blows and generating one’s own strikes, leads to deviations in homeostatic state at the cellular, tissue, and systemic levels (Finlay et al., 2020; Finlay et al., 2023; Ostapiuk-Karolczuk et al., 2025; Slimani et al., 2017). In this context, the period of reduced volume while maintaining intensity and competitive readiness is not merely a time of passive rest, but a vital, planned phase of manipulating training volume and intensity aimed at dissipating accumulated fatigue while simultaneously maintaining and maximizing physiological adaptations gained during the preparatory macrocycle (Uddin et al., 2020; Stone et al., 2025). The phenomenon of supercompensation, which is the ultimate goal of this phase, is characterized by a series of changes in the neuroendocrine system and immune profile. The application of ATR-FTIR spectroscopy to biofluids has been proposed as a method for the rapid characterization of exercise-related changes (Al-Kelani and Buthelezi, 2024; Bel’skaya et al., 2021; Valente et al., 2025). Whether such measurements may eventually contribute to assessing an athlete’s readiness to return to competitive performance remains an open question and cannot be determined from the present single-case study. Accordingly, the discussion below presents possible interpretations of the observed pattern, together with alternative explanations.

Boxing effort generates a specific type of musculoskeletal damage referred to as exercise-induced muscle damage (Obmiński et al., 2024). This process is driven by a dual pathway: mechanical stress, such as the eccentric work of the lower limbs and torso muscles during the delivery and cushioning of blows, and metabolic stress—the accumulation of hydrogen ions, inorganic phosphate, and a drop in pH that disrupts the functioning of calcium pumps (McCabe et al., 2023). The primary, clinically useful marker of this phenomenon is the intracellular cytosolic enzyme—CK, which catalyzes the reversible transfer of a phosphate group from phosphocreatine to ADP, conditioning ATP resynthesis in the working muscle (McCabe et al., 2023).

Data analysis showed a decrease in CK activity from 495 U/L on the first day of monitoring to 183 U/L on the day before the fight. The baseline value, though elevated relative to norms for the non-training population (typically up to approximately 190 U/L), is typical for elite combat sports athletes in the load accumulation phase (Tota and Wiecha, 2022). In sports such as boxing or MMA, the potentially high dynamics of fighting and training cause Z-line streaming in sarcomeres and the destabilization of structural proteins such as desmin or dystrophin (Brancaccio et al., 2010; Finlay et al., 2023). The consequence is an increase in sarcolemma permeability and the efflux of macromolecules, including CK, Mb, and LDH, into the interstitial space and from there, via lymphatic vessels, into the bloodstream (Brancaccio et al., 2010). A coefficient of variation of 37.6% for CK during the analyzed period highlights the dynamics of repair processes. The high muscle mass of a heavyweight athlete predisposes them to larger enzyme releases at the same relative load. CK fell by 63% between the first and final collection. The reduction is consistent with the process of decreasing myocellular damage and progressive cell membrane repair during tapering (McCabe et al., 2023). This interpretation is offered as a possibility rather than a demonstrated effect, since no formal test of the between-day differences was performed. In the case of CK, the resting concentration in an athlete of this body mass can vary several-fold between days independently of any change in load (Mougios, 2007). A causal attribution of the decline to the reduction in training volume would require repeated observations in multiple athletes with concurrently recorded load.

Muscle fiber damage is accompanied by an inflammatory response (Kılıc et al., 2019). The breakdown of cellular structures releases damage-associated molecular patterns (DAMPs) into the extracellular environment, which activate macrophages and neutrophils. These cells, infiltrating the damaged tissue to phagocytose necrotic debris, secrete a range of pro-inflammatory cytokines, including TNF-α, interleukin-1 beta (IL-1β), and IL-6 (Hacker et al., 2021; Lee, 2026). IL-6 acts as a primary endocrine mediator, stimulating hepatocytes in the liver to synthesize acute-phase proteins, among which CRP plays a dominant role (Hacker et al., 2021; Kılıc et al., 2019).

The decrease in CRP from 0.27 to 0.13 mg/dL recorded in the study is a positive indicator. It should be emphasized that in sports medicine, even small oscillations within the so-called laboratory norm (usually below 0.5 mg/dL) carry significant physiological implications. The positive, although not statistically significant, correlation between CRP and CK (*r* = 0.741; p = 0.152) is compatible with, but does not demonstrate, a link between the inflammatory state and mechanical muscle tissue damage rather than a latent infection (Ghoul et al., 2019; Kozioł et al., 2020). A persistent, elevated level of CRP in the pre-competition period would be an alarm signal, indicating a lack of adaptation, a possible transition into overtraining, and an increased risk of immunosuppression, which could result in a drop in performance and susceptibility to upper respiratory tract infections (Kozioł et al., 2020).

Studying combat sports athletes based on their endocrine profile provides key information about their ability to tolerate and adapt to extreme psycho-physical loads (Mondal et al., 2025). The hypothalamus–pituitary–adrenal (HPA) axis, responsible for cortisol production, and the hypothalamus–pituitary–gonadal (HPG) axis, regulating testosterone synthesis, constitute two antagonistic systems (Mondal et al., 2025; Tait et al., 2022). Changes in the concentrations of these hormones, and especially their mutual proportions, form the basis for monitoring and diagnostics in professional-level sports (Mondal et al., 2025).

Cortisol functions as an adaptive hormone, mobilizing energy substrates by increasing gluconeogenesis in the liver, proteolysis in skeletal muscles, and lipolysis in adipose tissue. From a textbook physiological perspective, reducing training volume and intensity during the “tapering” period should result in a clear drop in the concentration of this hormone (Liu et al., 2025). However, in the case discussed, the cortisol level remained stable throughout the entire 13 days (oscillating within a narrow range of 426–481 nmol/L), showing a low coefficient of variation (CV% = 4.4%). The lack of a drop in cortisol concentration in elite boxers before an important fight is not a symptom of poor recovery, but may manifest as a phenomenon known in sports psychophysiology as anticipatory stress (Tait et al., 2022). High-ranking sporting events trigger strong activation of the sympathetic nervous system and the HPA axis due to increasing psychological pressure, visualization of the upcoming confrontation, and fear of failure (Tait et al., 2022). The cerebral cortex and amygdala send signals to the hypothalamus, which releases corticotropin-releasing hormone (CRH), stimulating the anterior pituitary gland to secrete adrenocorticotropic hormone (ACTH), which in turn sustains cortisol synthesis in the zona fasciculata of the adrenal cortex (Kılıc et al., 2019). CRP and CK showed a positive correlation across the five measurement points (*r* = 0.741); however, this finding should be interpreted with considerable caution because the correlation was based on only five observations. The wide 95% confidence interval (−0.39 to 0.98) and the unadjusted *p*-value of 0.152 indicate substantial uncertainty around this estimate. The observed association may therefore reflect both concurrent changes in CRP and CK related to muscle damage and independent fluctuations in the two markers. However, it does not allow us to conclude that the observed changes in CRP were secondary to muscle damage. In addition, the study did not include screening for infection or other potential causes of inflammation that could have helped distinguish between these possible mechanisms. Persistently elevated CRP concentrations during the pre-competition period have previously been described as a potential warning sign of incomplete physiological adaptation (Kozioł et al., 2020); however, the present data neither confirm this association nor provide additional evidence in support of it.

Testosterone was higher at the final collection than at the first (15.23 to 17.55 nmol/L), but the ordinary least-squares slope across all five collections was not distinguishable from zero (0.069 nmol·L^-1^·day^-1^, 95% CI −0.412 to 0.550, *p* = 0.679). Recovery of HPG-axis function following a reduction in load is a phenomenon described in the subject literature (Pritchard et al., 2019) and would be consistent with the direction of this change, but these data do not demonstrate supercompensation. Heavy resistance and endurance training in the phase preceding the tapering period usually causes transient suppression of luteinizing hormone (LH) secretion, resulting in decreased testosterone synthesis in Leydig cells (Pritchard et al., 2019). Reducing training volume allows for a return to, and then an exceeding of, baseline values. This increase is important for the regeneration of muscle fibers, as testosterone diffuses through the sarcolemma and binds to the androgen receptor in the cytosol, and the resulting complex moves to the cell nucleus, where it acts as a transcription factor, increasing the expression of genes responsible for the synthesis of myosin and actin (Pritchard et al., 2019). The consequence of cortisol stabilization combined with a simultaneous increase in testosterone is the occurrence of gradual favorable changes for the T-to-C ratio, which, in sports, is considered a key barometer of the balance between anabolic and catabolic processes (Mondal et al., 2025). The T-to-C ratio (×100) was 3.4 at the first collection and 4.1 at the last. Because the ratio compounds the variability of both hormones (Sollberger and Ehlert, 2016), it is less stable than either component separately.

High-intensity sport, involving an increase in oxygen consumption by mitochondria, generates elevated amounts of reactive oxygen species (ROS) and reactive nitrogen species (RNS), such as superoxide radicals, hydrogen peroxide, or hydroxyl radicals. Although endogenous ROS play a positive signaling role in moderate amounts—for example, by inducing mitochondrial biogenesis or the expression of antioxidant enzymes—their excess leads to the phenomenon of oxidative stress, damaging cellular structures (Kamati et al., 2025; Li et al., 2025; Mallett and McGrath, 2025).

Lipid peroxidation is the process of oxidizing polyunsaturated fatty acids that are part of cell membranes, leading to a decrease in their fluidity, disruption of membrane protein functions, and ultimately cell lysis (Lee et al., 2019). The primary marker of this process is malondialdehyde. Analysis of the studied boxer’s profile showed that the MDA level remained stable throughout the entire 13-day cycle, staying within narrow limits from 0.62 to 0.81 μmol/L (CV% = 10.1%). The lack of an increase in MDA concentration, despite continuing volume-reduced training units, may serve as evidence of the athlete’s body adaptation. Potentially, this means that the athlete’s antioxidant capacities were able to neutralize the produced portion of free radicals in real time before they could cause damage to the lipid architecture of the membranes (Kamati et al., 2025; Tshibangu, 2024).

Most of the parameters described above are routinely determined in analytical laboratories using expensive, time-consuming immunoenzymatic or colorimetric methods. An innovative aspect of the presented material is the use of ATR-FTIR as a “fingerprinting” method for biofluids for the parallel, simultaneous evaluation of selected markers (Lemes et al., 2016; Valente et al., 2025). FTIR technology involves irradiating a minute volume with infrared radiation. Organic molecules, depending on the mass of the atoms and the strength of the bonds between them, absorb quanta of energy at specific frequencies, leading to the excitation of their oscillatory vibrations (Bel’skaya et al., 2021). After passing through a Michelson interferometer and applying a mathematical Fourier transform, a unique transmission or absorbance spectrum is obtained, which is a summary reflection of the biochemical composition of the tissue (Bel’skaya et al., 2021; Valente et al., 2025). From the perspective of sports physiology, FTIR provides an image of the metabolome almost instantaneously, which, in the conditions of pre-competition training camps, can constitute a major advantage in athlete preparation.

Plasma cortisol showed a positive correlation with the intensity of the band at 3,009 cm^−1^ (*r* = 0.89). The spectral region in the range of 3,000–3,015 cm^−1^ is typically assigned to the =C–H stretching vibrations of cis-unsaturated acyl chains, characteristic of unsaturated fatty acids and cholesterol esters present in circulating lipoproteins (Talari et al., 2017). At a plasma concentration of approximately 450 nmol/L, however, cortisol is present at far too low an abundance to contribute measurably to the intensity of this band. One possible explanation could be an indirect relationship, in which cortisol stimulates lipolysis, and changes in cortisol exposure may co-occur with changes in the circulating pool of unsaturated fatty acids. This mechanism, however, was not verified in the present study. Its confirmation would require, at a minimum, the simultaneous determination of the plasma fatty acid profile and a demonstration that the observed spectral changes truly reflect alterations in this profile. This hypothesis also fails to explain why the relationship should be positive under resting conditions across five consecutive morning samples. Considering that the observed correlation did not survive correction for multiple testing, the most cautious interpretation is that it constitutes an interesting observation requiring independent replication. This result does not justify treating FTIR as a direct method for determining cortisol or as a substitute for conventional measurement of its concentration.

TGlu and RGlu showed a negative correlation with the bands at 671, 699, and 721 cm^−1^ (|*r*| = 0.89–0.96), with *R*^2^ values ranging from 0.80 to 0.92. The bands at 671 and 699 cm^-1^ are typically assigned to out-of-plane C–H deformation vibrations in aromatic rings, whereas the band at 721 cm^−1^ is primarily associated with thymine (Movasaghi et al., 2008; Talari et al., 2017). One possible, yet unconfirmed hypothesis is that the relationship between glutathione status and absorbance in this region may be partly related to protein S-glutathionylation and deglutathionylation processes. Glutathione protects protein thiol groups from irreversible oxidation by forming mixed disulfides with cysteine residues (Kuleshova et al., 2023). If an increase in glutathione synthesis alongside a reduction in training load is accompanied by enhanced deglutathionylation of plasma proteins, this could potentially affect the local environment of sulfur-containing and aromatic residues, and consequently the FTIR spectrum characteristics. However, this hypothesis was not directly verified in the present study. The interpretation of these results is further constrained by the analytical quality of the bands themselves. All three signals were weak, located in the region with the lowest signal-to-noise ratio in the analyzed spectra. Therefore, the observed relationships should be treated primarily as hypotheses requiring confirmation in studies with a larger number of observations.

### 4.1 Limitations of the study

The study comprised one participant and five biological observations. Eight spectra recorded for each sample constituted technical replicates obtained under identical analytical conditions; they illustrate within-sample visual consistency but do not increase the number of independent biological observations and do not allow repeatability or reproducibility to be established across independent runs or measurement days. Because of the inclusion of only a single participant, it was not possible to estimate inter-individual variability or incorporate it into statistical models. Consequently, nominal degrees of freedom and the resulting *p*-values may overstate the strength of the evidence. A total of 264 correlations between spectral bands and biochemical parameters, as well as 66 correlations among the biochemical parameters themselves, were calculated. Six of the 264 spectral–biochemical coefficients reached an uncorrected value of *p* < 0.05, whereas with this number of tests approximately 13 nominal results could be expected purely by chance. None of the obtained coefficients survived Benjamini–Hochberg correction. Correlation results should therefore be treated primarily as exploratory. The spectral bands included in the regression models were selected based on their correlations with biochemical parameters within the same five observations that were subsequently used to fit and evaluate the models. Such a predictor selection procedure can lead to an inflation of *R*^2^ values and causes them to represent model fit to the analyzed sample. Training load was determined based on prospectively recorded training volume, internal load assessed via session RPE, and sparring exposure. Objective measures of external load were not included, and dietary intake, fluid balance, sleep duration, and sleep quality were not systematically recorded. Given that the observed biochemical changes are discussed in the context of reduced training load, alternative explanations for these changes cannot be excluded. All biochemical assays and all spectra were acquired within a single analytical run. This limits the occurrence of between-run differences and eliminates potential inter-run drift but, at the same time, means there is no independent replication of the entire analytical process. Samples underwent a single freeze–thaw cycle prior to measurement, which may have been of particular significance for glutathione fractions, as RGlu is an unstable compound. The oxidized fraction was not assayed directly, but was calculated based on other measurements; therefore, its variability additionally reflects the variability of the two directly assayed fractions. The very high coefficient of variation for the RGlu-to-OxGlu ratio (133.9%) resulted largely from a single low oxidized glutathione value. The analysis was restricted to the intensities of 22 pre-defined spectral bands. More advanced multivariate approaches, such as principal component analysis combined with discriminant methods, would enable the analysis of the full spectral profile and could constitute appropriate analytical frameworks in future studies. However, applying such methods to the current dataset, comprising only five biological observations, would entail a very high risk of fitting them to noise. Their utilization should therefore be considered only in studies involving an adequately large sample.

## 5 Conclusions

Repeated ATR-FTIR measurements of blood plasma were technically feasible throughout a 13-day pre-competition period in a professional heavyweight boxer. Eight technical replicates were acquired from each plasma sample under identical analytical conditions, and the resulting spectra were visually consistent (**Supplementary Figures S1**–**S5**); this illustrates within-sample visual consistency and does not establish quantitative repeatability or reproducibility across independent runs, operators, instruments, or measurement days. Demonstrating the technical feasibility of this workflow under realistic pre-competition conditions, with a sample volume of just 4 µL, constitutes the main contribution of this study. A screening analysis encompassing 264 correlations identified four spectral regions: 671, 699, and 721 cm^−1^ co-varying with TGlu and RGlu, and 3,009 cm^−1^ co-varying with cortisol. They are presented solely to direct subsequent hypothesis-testing studies toward specific spectral regions.

## Data Availability

The original contributions presented in the study are included in the article/**Supplementary Material**. Further inquiries can be directed to the corresponding author.

## References

[B1] Al-Kelani M. Buthelezi N. (2024). Advancements in medical research: Exploring Fourier Transform Infrared (FTIR) spectroscopy for tissue, cell, and hair sample analysis. Skin Res. Technol. 30, e13733. doi: 10.1111/srt.13733 38887131 PMC11182784

[B51] Barth A. (2007). Infrared spectroscopy of proteins. Biochim. Biophys. Acta 1767, 1073–1101. doi: 10.1016/j.bbabio.2007.06.004 17692815

[B2] Bel’skaya L. V. Sarf E. A. Solomatin D. V. (2021). Application of FTIR spectroscopy for quantitative analysis of blood plasma: A preliminary study. Diagnostics 11, 2391. doi: 10.3390/diagnostics11122391 34943626 PMC8700755

[B3] Brancaccio P. Lippi G. Maffulli N. (2010). Biochemical markers of muscular damage. Clin. Chem. Lab. Med. 48, 757–767. doi: 10.1515/CCLM.2010.179 20518645

[B4] Brancaccio P. Maffulli N. Limongelli F. M. (2007). Creatine kinase monitoring in sport medicine. Br. Med. Bull. 81–82, 209–230. doi: 10.1093/bmb/ldm014 17569697

[B5] Brini S. Ahmaidi S. Bouassida A. (2020). Effects of passive versus active recovery at different intensities on repeated sprint performance and testosterone/cortisol ratio in male senior basketball players. Sci. Sports 35, e142–e147. doi: 10.1016/j.scispo.2019.07.01542574925

[B6] Capranica L. Condello G. Tornello F. Iona T. Chiodo S. Valenzano A. . (2017). Salivary alpha-amylase, salivary cortisol, and anxiety during a youth taekwondo championship: An observational study. Medicine 96, e7272. doi: 10.1097/MD.0000000000007272 PMC5515742 28700470

[B7] Clarkson P. M. Kearns A. K. Rouzier P. Rubin R. Thompson P. D. (2006). Plasma creatine kinase levels and renal function measures in exertional muscle damage. Med. Sci. Sports Exercise 38, 623–627. doi: 10.1249/01.mss.0000210192.49210.fc 16679975

[B8] Daly W. Seegers C. A. Rubin D. A. Dobridge J. D. Hackney A. C. (2005). Relationship between stress hormones and testosterone with prolonged endurance exercise. Eur. J. Appl. Physiol. 93, 375–380. doi: 10.1007/s00421-004-1223-1 15618989

[B9] De Luccia T. P. B. (2016). Use of the testosterone/cortisol ratio variable in sports. Open Sports Sci. J. 9, 104–113. doi: 10.2174/1875399x01609010104

[B10] Ficarra G. Caccamo D. Rottura M. Bitto A. Trimarchi F. Di Mauro D. (2023). Testosterone:cortisol ratio as a predictor of podium in adolescent rowing athletes. Heliyon 9, e22315. doi: 10.1016/j.heliyon.2023.e22315 38053894 PMC10694314

[B11] Finlay M. J. Greig M. McCarthy J. Page R. M. (2020). Physical response to pad- and bag-based boxing-specific training modalities. J. Strength Conditioning Res. 34, 1052–1061. doi: 10.1519/JSC.0000000000002928 30358696

[B12] Finlay M. J. Greig M. Page R. M. Bridge C. A. (2023). Acute physiological, endocrine, biochemical and performance responses associated with amateur boxing: A systematic review with meta-analysis. Eur. J. Sport Sci. 23, 774–788. doi: 10.1080/17461391.2022.2063072 35380916

[B13] Ghoul N. Tabben M. Miarka B. Tourny C. Chamari K. Coquart J. (2019). Mixed Martial Arts induces significant fatigue and muscle damage up to 24 hours post-combat. J. Strength Conditioning Res. 33. doi: 10.1519/JSC.0000000000002078 28658085

[B14] Hacker S. Reichel T. Hecksteden A. Weyh C. Gebhardt K. Pfeiffer M. . (2021). Recovery-stress response of blood-based biomarkers. Int. J. Environ. Res. Public Health 18, 5776. doi: 10.3390/ijerph18115776 34072201 PMC8199256

[B15] Hagstrom A. D. Shorter K. A. (2018). Creatine kinase, neuromuscular fatigue, and the contact codes of football: A systematic review and meta-analysis of pre- and post-match differences. Eur. J. Sport Sci. 18, 1234–1244. doi: 10.1080/17461391.2018.1480661 29870313

[B16] Isaacs A. W. Macaluso F. Smith C. Myburgh K. H. (2019). C-Reactive protein is elevated only in high creatine kinase responders to muscle damaging exercise. Front. Physiol. 10, 86. doi: 10.3389/fphys.2019.00086 PMC6378920 30804809

[B17] Jiménez M. Aguilar R. Alvero-Cruz J. R. (2012). Effects of victory and defeat on testosterone and cortisol response to competition: Evidence for same response patterns in men and women. Psychoneuroendocrinology 37, 1577–1581. doi: 10.1016/j.psyneuen.2012.02.011 22429747

[B18] Kamati O. V. Bragagna L. Bester D. J. Wagner K. H. Stürmer V. Gassner M. . (2025). A standardised exercise protocol to induce oxidative stress in humans: Validation with a dietary polyphenol intervention. Nutrients 17, 2971. doi: 10.3390/nu17182971 41010497 PMC12472591

[B19] Kılıc Y. Cetin H. N. Sumlu E. Pektas M. B. Koca H. B. Akar F. (2019). Effects of boxing matches on metabolic, hormonal, and inflammatory parameters in male elite boxers. Medicina 55, 288. doi: 10.3390/medicina55060288 31216765 PMC6630693

[B20] Kozioł K. Zebrowski J. Betlej G. Bator E. Czarny W. Bajorek W. . (2020). Changes in γH2AX and H4K16ac levels are involved in the biochemical response to a competitive soccer match in adolescent players. Sci. Rep. 10, 14481. doi: 10.1038/s41598-020-71436-6 32879387 PMC7468116

[B21] Kuleshova I. D. Zaripov P. I. Poluektov Y. M. Anashkina A. A. Kaluzhny D. N. Parshina E. Y. . (2023). Changes in hemoglobin properties in complex with glutathione and after glutathionylation. Int. J. Mol. Sci. 24, 13557. doi: 10.3390/ijms241713557 37686361 PMC10487563

[B22] Lee H. S. (2026). Effects of rapid weight loss on the immune system in combat sports athletes: A systematic review. Int. J. Mol. Sci. 27, 508. doi: 10.3390/ijms27010508 41516380 PMC12787199

[B23] Lee S.-H. Scott S. D. Pekas E. J. Lee J.-G. Park S.-Y. (2019). Improvement of lipids and reduction of oxidative stress with octacosanol after taekwondo training. Int. J. Sports Physiol. Perform. 14, 1297–1303. doi: 10.1123/ijspp.2018-0704 30958045

[B24] Lemes L. C. Caetano Júnior P. C. Strixino J. F. Aguiar J. Raniero L. (2016). Analysis of plasma cortisol levels by Fourier Transform Infrared Spectroscopy for diagnosis of stress in athletes. Res. Biomed. Eng. 32, 293–300. doi: 10.1590/2446-4740.0121641099703

[B25] Li R. Huang S. Liu S. Liu Q. Chen X. (2025). Assessment of nanoplastic-induced disruption in cellular glutathione metabolism using a bubble-assisted photothermal capture SERS sensor. Anal. Chem. 97, 19177–19185. doi: 10.1021/acs.analchem.5c02791 40877114

[B26] Liu Y. Liu X. Hu Y. Gao F. Yu W. Cheng F. (2025). Evaluating untargeted metabolomics pipelines for sports nutrition research: A review. Anal. Methods 17, 8847–8864. doi: 10.1039/d5ay01484k 41186329

[B27] Mallett G. S. McGrath K. (2025). Effect of endurance exercise on markers of oxidative stress: A systematic review. J. Sci. Sport Exercise 7, 175–196. doi: 10.1007/s42978-024-00305-930311153

[B28] Markus I. Constantini K. Hoffman J. R. Bartolomei S. Gepner Y. (2021). Exercise-induced muscle damage: Mechanism, assessment and nutritional factors to accelerate recovery. Eur. J. Appl. Physiol. 121, 969–992. doi: 10.1007/s00421-020-04566-4 33420603

[B29] McCabe D. Martin D. McMahon G. (2023). Training load is correlated with changes in creatine kinase and wellness over a 12-week multi-stage preparatory training block for a major competition in international boxers. Physiologia 3, 585–597. doi: 10.3390/physiologia304004330654563

[B30] Mondal S. Hathi D. K. Bhattacharya S. Kalra S. (2025). The testosterone: Cortisol ratio - A tool with practical use and research potential in endocrinology. Indian J. Endocrinol. Metab. 29. doi: 10.4103/ijem.ijem_85_25 PMC12604835 41229716

[B31] Mougios V. (2007). Reference intervals for plasma creatine kinase in athletes. Br. J. Sports Med. 41, 674–678. doi: 10.1136/bjsm.2006.034041 17526622 PMC2465154

[B32] Movasaghi Z. Rehman S. Rehman I. U . (2008). Fourier transform infrared (FTIR) spectroscopy of biological tissues. Appl. Spectrosc. Rev. 43, 134–179. doi: 10.1080/05704920701829043

[B33] Obmiński Z. Crewther B. T. Cook C. J. (2024). Disentangling the dynamic interplay between muscle damage and energetics in male boxers during a short training block. Biol. Sport 41, 69–75. doi: 10.5114/biolsport.2024.127383 38188101 PMC10765437

[B34] Ostapiuk-Karolczuk J. Dziewiecka H. Bojsa P. Cieślicka M. Zawadka-Kunikowska M. Wojciech K. . (2025). Biochemical and psychological markers of fatigue and recovery in mixed martial arts athletes during strength and conditioning training. Sci. Rep. 15, 24234. doi: 10.1038/s41598-025-09719-z 40624253 PMC12234679

[B35] Papacosta E. Nassis G. P. Gleeson M. (2016). Salivary hormones and anxiety in winners and losers of an international judo competition. J. Sports Sci. 34, 1281–1287. doi: 10.1080/02640414.2015.1111521 26584022

[B37] Pritchard H. J. Barnes M. J. Stewart R. J. Keogh J. W. McGuigan M. R. (2019). Higher- versus lower-intensity strength-training taper: Effects on neuromuscular performance. Int. J. Sports Physiol. Perform. 14, 458–463. doi: 10.1123/ijspp.2018-0489 30204523

[B38] Puiu A. A. Radke S. Votinov M. Habel U. Herpertz-Dahlmann B. Turetsky B. . (2019). Plasma testosterone and cortisol concentrations after single-dose administration of 100-mg transdermal testosterone in healthy men. Front. Pharmacol. 10, 1397. doi: 10.3389/fphar.2019.01397 PMC6881786 31824320

[B40] Salvador A. Suay F. González-Bono E. Serrano M. A. (2003). Anticipatory cortisol, testosterone and psychological responses to judo competition in young men. Psychoneuroendocrinology 28, 364–375. doi: 10.1016/S0306-4530(02)00028-8 12573302

[B41] Slimani M. Chaabene H. Miarka B. Franchini E. Chamari K. Cheour F. (2017). Kickboxing review: Anthropometric, psychophysiological and activity profiles and injury epidemiology. Biol. Sport 34, 185–196. doi: 10.5114/biolsport.2017.65338 28566813 PMC5424459

[B42] Sollberger S. Ehlert U. (2016). How to use and interpret hormone ratios. Psychoneuroendocrinology 63, 385–397. doi: 10.1016/j.psyneuen.2015.09.031 26521052

[B43] Souza R. A. Beltran O. A. B. Zapata D. M. Silva E. Freitas W. Z. Junior R. V. . (2019). Heart rate variability, salivary cortisol and competitive state anxiety responses during pre-competition and pre-training moments. Biol. Sport 36, 39–46. doi: 10.5114/biolsport.2018.78905 30899138 PMC6413577

[B44] Stone M. J. Knight C. J. Hall R. Cropley B. Shearer C. Nicholas R. . (2025). Elite swimmers’ and coaches’ understanding and psychological experience of taper: A multi-phase qualitative investigation. J. Appl. Sport Psychol. 38(3), 365–390. doi: 10.1080/10413200.2025.250763637339054

[B45] Tait J. L. Drain J. R. Corrigan S. L. Drake J. M. Main L. C. (2022). Impact of military training stress on hormone response and recovery. PloS One 17, e0265121. doi: 10.1371/journal.pone.0265121 35271678 PMC8912193

[B39] Talari A. C. S. Martinez M. A. G. Movasaghi Z. Rehman S. Rehman I. U . (2017). Advances in Fourier transform infrared (FTIR) spectroscopy of biological tissues. Appl. Spectrosc. Rev. 52, 456–506. doi: 10.1080/05704928.2016.1230863

[B47] Tota Ł. Piotrowska A. Pałka T. Morawska M. Mikuľáková W. Mucha D. . (2019). Muscle and intestinal damage in triathletes. PloS One 14, e0210651. doi: 10.1371/journal.pone.0210651 30657773 PMC6338373

[B46] Tota Ł.M. Wiecha S. S. (2022). Biochemical profile in mixed martial arts athletes. PeerJ 10, e12708. doi: 10.7717/peerj.12708 35047233 PMC8759358

[B48] Totsuka M. Nakaji S. Suzuki K. Sugawara K. Sato K. (2002). Break point of plasma creatine kinase release after endurance exercise. J. Appl. Physiol. 93, 1280–1286. doi: 10.1152/japplphysiol.01270.2001 12235026

[B49] Tshibangu A. M. N. (2024). Boxing practitioners physiology review: 3. Dietary supplementation, weight control, recovery and altitude. Open J. Mol. Integr. Physiol. 14, 1–29. doi: 10.4236/ojmip.2024.141001

[B36] Uddin N. Tallent J. Waldron M . (2020). Physiological and perceptual responses to a five-week pre-event taper in professional mixed martial arts athletes. J. Sport Exerc. Sci. 4, 90–99. doi: 10.36905/jses.2020.02.04

[B50] Valente P. A. Mota S. I. Teixeira A. Ferreiro E. Sarmento H. Cipriano I. . (2025). Fourier Transform Infrared (FTIR) spectroscopy as a tool to characterize exercise and physical activity: A systematic review. Sports Med. 55, 459–472. doi: 10.1007/s40279-024-02139-5 39565518 PMC11946944

