# Supplementary material for: Application of FTIR to detect changes in blood plasma during a pre-competition period in a professional heavyweight boxer
Source: Front Physiol. 2026 Oct 19;17:1845333. doi: 10.3389/fphys.2026.1845333 (PMC13724598; doi:10.3389/fphys.2026.1845333)
Supplement: Supplementary file 1 [file SupplementaryFile1.docx]

**Figure S1.** All 8 FTIR spectra obtained from sample collected at 10.05.2024.

**Figure S2.** All 8 FTIR spectra obtained from sample collected at 13.05.2024.

**Figure S3.** All 8 FTIR spectra obtained from sample collected at 17.05.2024.

**Figure S4.** All 8 FTIR spectra obtained from sample collected at 20.05.2024.

**Figure S5.** All 8 FTIR spectra obtained from sample collected at 23.05.2024.
